# Development of a transformation system for *Aspergillus sojae* based on the *Agrobacterium tumefaciens*-mediated approach

**DOI:** 10.1186/s12866-014-0247-x

**Published:** 2014-09-25

**Authors:** Rodrigo Mora-Lugo, Judith Zimmermann, Amira M Rizk, Marcelo Fernandez-Lahore

**Affiliations:** Jacobs University Bremen gGmbH, Campus Ring 1, 28759 Bremen, Germany; Max Planck Institute for Marine Microbiology, Celsiusstrasse 1, 28359 Bremen, Germany

**Keywords:** *Ble* gene, Enhanced green fluorescent protein (EGFP), Filamentous fungus, Transfer DNA (T-DNA), Western blot

## Abstract

**Background:**

*Aspergillus sojae* has been an important filamentous fungus in Biotechnology due to its use in diverse fermentative processes for the production of various food products. Furthermore, this fungus is a common expression system for the production of enzymes and other metabolites. The availability of molecular genetic tools to explore its biology is thus of big interest. In this study, an *Agrobacterium tumefaciens*-mediated transformation (ATMT) system for *A. sojae* was developed and its applicability evaluated.

**Results:**

The donor plasmid named pRM-eGFP was constructed for ATMT of *A. sojae*. This plasmid contains the *ble* and *egfp* genes in its transfer DNA element (T-DNA) to confer phleomycin resistance and express the enhanced green fluorescent protein (EGFP) in *A. sojae*, respectively. *Agrobacterium tumefaciens* (LBA4404) harboring the donor plasmid and *A. sojae* (ATCC 20235) were co-cultured under diverse conditions to achieve ATMT. The maximum number of transformed fungi was obtained after three days of co-culturing at 28°C, and selection with 50 μg/ml phleomycin. Polymerase chain reaction (PCR), fluorescence microscopy and Western Blot analysis for EGFP expression confirmed successful genomic integration of the T-DNA element in *A. sojae*. The T-DNA was mitotically stable in approximately 40% of the fungal transformants after four generations of sub-culturing under phleomycin pressure.

**Conclusion:**

We successfully established a new ATMT protocol for *A. sojae*. This transformation system should enable further protein expression studies on this filamentous fungus.

## Background

*Aspergillus sojae* is a filamentous fungus and a well-known *koji* mold. Like *Aspergillus oryzae*, *A. sojae* is widely used for the production of oriental food and beverage products like soy sauce, sake (rice wine) and miso (soybean paste) [1]. Moreover, these fungi have the ability to secrete large amounts of hydrolytic enzymes. Diverse homologous and heterologous proteins have been expressed in *A. sojae* [2], and its potential for the production of commercially important enzymes like pectinases [3], mannanases [4] or glutaminases [5] has been demonstrated. Its GRAS status (generally recognized as safe) has been advantageous over other toxigenic (aflatoxin-producing) filamentous fungi for many bioprocess applications [6]. Thus, the availability of molecular genetic tools to explore its biology is of big interest.

In the last years, *Agrobacterium tumefaciens*-mediated transformation (ATMT) has become a common technique for selected filamentous fungi [7]. ATMT is based on the capacity of *A. tumefaciens* to transfer part of its DNA (T-DNA), contained in the tumor-inducing (Ti) plasmid, to the host cell. Such T-DNA, delimited by imperfect 25-base pair repeats, called the right and left border sequences (RB and LB, respectively), is typically randomly inserted in the host genome as a single copy [8]. Until now, a wide variety of different fungal species have been transformed using this approach, with *Aspergillus terreus* [9] and *Aspergillus carbonarius* [10] as some of the last examples. However, to our knowledge, the applicability of ATMT in an *A. sojae* strain has so far not been tested.

In this study, we set up an ATMT system for *A. sojae*, using the *ble* gene as an antibiotic selector marker for recombinant fungi. The effectiveness of this method was measured and further validated as a genetic molecular tool for *A. sojae*.

## Methods

### Microorganisms

*A. sojae* ATCC 20235 (*A. sojae*) was grown at 28°C on potato dextrose agar (PDA) plates until conidiation (5–8 days). Spores were harvested using 0.02% (*v/v*) Tween 80, and filtered through cotton to remove hyphae. The spore concentration was determined using a hemocytometer (Thoma, Germany).

Top 10 *Escherichia coli* cells (Invitrogen, USA) were used as a host for all DNA manipulations. DNA plasmids were isolated from Luria-Bertani (LB) overnight cultures supplemented with 100 μg/ml streptomycin or 50 μg/ml kanamycin as required, using the “NucleoSpin Plasmid” commercial kit (Macherey-Nagel, Germany).

*Agrobacterium tumefaciens,* strain LBA4404 (ElectroMAX™, Invitrogen, USA) was used as T-DNA donor for fungal transformation of *A. sojae*.

### Phleomycin minimum inhibitory concentration for *A. sojae*

The sensitivity of *A. sojae* to phleomycin was assayed. Phleomycin is a glycopeptide antibiotic of the bleomycin family, which binds and intercalates DNA thus destroying the integrity of the double helix. A total amount of 10^5^*A. sojae*-spores was inoculated in minimal media agar plates (MM) (10 mM K_2_HPO_4_, 10 mM KH_2_PO_4_, 2.5 mM NaCl, 2 mM MgSO_4_°7H_2_O, 0.7 mM CaCl_2_, 9 μM FeSO_4_°7H_2_O, 4 mM (NH_4_)_2_SO_4_, 10 mM glucose and 2% (*w/v*) agar) with various antibiotic concentrations: 10, 25, 50, 100 μg/ml and no antibiotic. The plates were incubated at 29°C, and the appearance of fungal colonies was examined during 7 days.

### Construction of T-DNA donor vector

**Figure 1:**
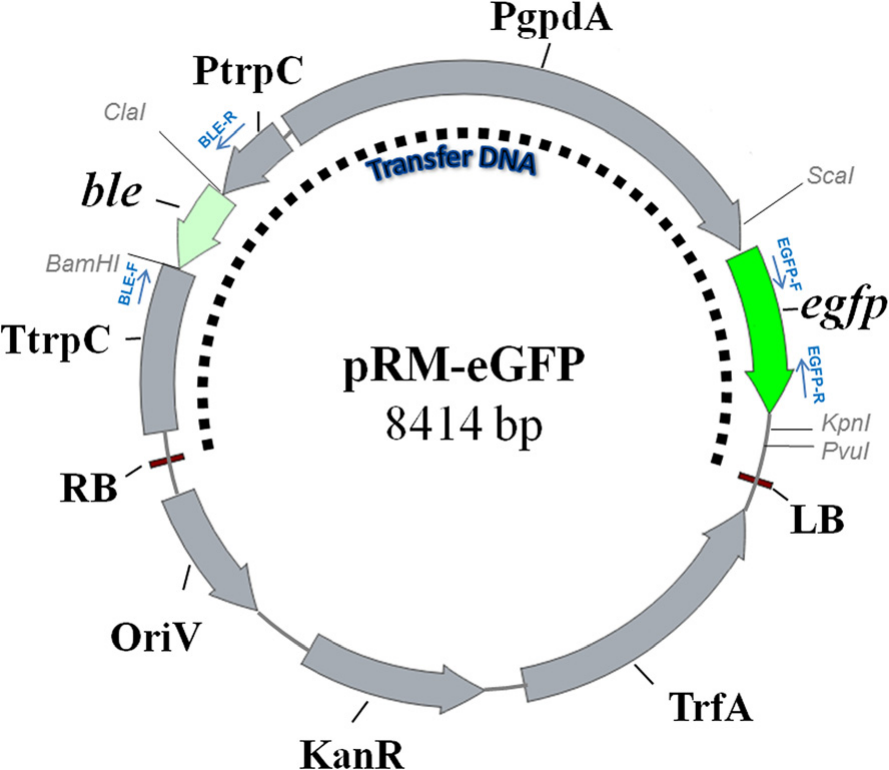
**The ATMT donor vector pRM-eGFP.** The transfer DNA region (T-DNA) consists of the phleomycin-resistance conferring gene (*ble*) which is under control of the *A. nidulants trpC* promoter (PtrpC) and *trpC* terminator (TtrpC). The enhanced green fluorescent protein (EGFP) reporter gene is under control of the constitutive *A. nidulants gpdA* promoter (PgpdA). The blue arrows indicate the target sites for the oligonucleotide primers BLE-F, BLE-R, EGFP-F and EGFP-R. OriV = replication origin; KanR = kanamycin resistance gene; TrfA = trans-acting gene trfA.

The *A. tumefaciens* transforming vector named pRM-eGFP (Figure 1), was designed to confer phleomycin resistance and express EGFP reporter protein in *A. sojae* (*ble* and *egfp* genes, respectively) through an ATMT procedure. The expression of both genes is driven by the strong constitutive *Aspergillus nidulants gpdA* and *trpC* promoters (PgpdA and PtrpC, respectively), extensively used for protein expression in *Aspergillus* species [11]. This vector was derived from the pRFHUE-eGFP vector [10] by replacement of the hygromycin B phosphotransferase gene (*hph*), with the phleomycin gene (*ble*). To construct the pRM-eGFP vector, a two-step subcloning strategy was followed. Firstly, multiple ClaI and BamHI sites in the pRFHUE-eGFP vector were deleted by amplifying a 2.5-kbp-length fragment from this vector with the primers ML1.3-F and ML1.4-R, which incorporates each a BssHII restriction site. This PCR product was digested and ligated by BssHII restriction site into the pRFHUE-eGFP vector, resulting in a 9068 bp plasmid with unique ClaI and BamHI sites delimitating the *hph* gene. In the second step, the *hph* gene was replaced by the *ble* gene using the ClaI and BamHI restriction sites. The *ble* gene was amplified from the pGAPZαA vector (Invitrogen) with the primers cla-F and bam-R, which incorporates ClaI and BamHI restriction sites (Table 1). Sequencing and restriction enzyme digestion analysis were carried out to verify correctly assembled plasmid. The resulting pRM-eGFP vector (Figure 1) was transformed into *A. tumefaciens* LBA4404 electrocompetent cells and selected on LB agar plates containing 100 μg/ml streptomycin and 50 μg/ml kanamycin. The oligonucleotide primer sequences and PCR conditions used are listed in Table 1.

**Table 1:** **Oligonucleotide primers and PCR conditions used in this study**

**Name/Sequence (5′-3′)**	**Function**	**PCR conditions**
ML1.3-F/TCCGGCGgcgcgcATCCTCTAGAAAGAAGGATTAC	Mutagenesis on pRFHUE-eGFP vector	**ID:** 94°C for 5 min.
ML1.3-F/TCCGGCGgcgcgcATCCTCTAGAAAGAAGGATTAC		**5-cycle:** 94°C for 15 sec, 60°C for 15 sec and 68°C for 3 min.
		**30-cycle:** 94°C for 15 sec and 68°C for 3 min.
		**FE:** 68°C for 5 min.
cla-F/GAGGAatcgatCC*ATG*GCCAAGTTG	Amplification of *ble* gene from pGAPZαA vector	**ID:** 94°C for 5 min.
		**30-cycle:** 94°C for 15 sec, 62°C for 15 sec and 68°C for 45 sec.
bam-R/TCggatccG*TCA*GTCCTGCTCCTCG		
		**FE:** 68°C for 5 min.
BLE-F/CGTTTTATTCTTGTTGACATGGAGC	Target *ble* gene contained on T-DNA	**ID:** 94°C for 5 min.
		**30-cycle:** 94°C for 15 sec, 55°C for 15 sec and 68°C for 45 sec.
BLE-R/TTGGGCTTGGCTGGAGCTAGTGGAG		
		**FE:** 68°C for 5 min.
EGFP-F/ACCTACGGCAAGCTGACCCTGAAGT	Target *egfp* gene contained on T-DNA	**ID:** 94°C for 5 min.
		**30-cycle:** 94°C for 15 sec, 60°C for 15 sec and 68°C for 45 sec.
EGFP-R/TGTACAGCTCGTCCATGCCGAGAGT		
		**FE:** 68°C for 5 min.

Lowercase letters gcgcgc, atcgat and ggatcc indicate restriction sites BssHII, ClaI and BamHI respectively. The italicized sequences *ATG* and *TCA*, correspond to the star and stop codon, respectively. ID: initial denaturation; FE: final elongation.

### *Agrobacterium tumefaciens*-mediated transformation

The ATMT was carried out following indications from previous reports [8,12]. The *A. tumefaciens* LBA4404 strain harboring the T-DNA binary vector pRM-eGFP was grown overnight at 28°C on a rotatory shaker (Innova 4000, New Brunswick Scientific, USA) at 200 rpm in 5 ml of LB broth supplemented with 50 μg/ml streptomycin and 50 μg/ml kanamycin. The overnight culture was centrifuged for 10 min and 3,200 × *g* at room temperature and the pellet was washed twice with one volume of fresh induction medium (IM) (MM plus 0.5% (*v/v*) glycerol, 200 μM acetosyringone (AS) and 40 mM 2-(N-morpholino)ethanesulfonic acid (MES), pH 5.3). Finally, the bacterial pellet was resuspended in 10 ml of IM to an OD_600_ of 0.1. The bacterial cells were further grown under the same conditions to an OD_600_ of 0.5–0.6. In parallel, a 10^5^*A. sojae*-conidia/ml water suspension was prepared with fresh spores harvested from a PDA agar plate as described above. For the co-cultivation of *A. tumefaciens* and *A. sojae*, 100 μl of each the bacterial and fungal suspensions, were spread evenly on a 50 mm filter paper laid on top of 25 ml of an IM agar plate (IM plus 1.8% (*w/v*) agar) and incubated in the dark at 28°C. The co-culture growth from this plate was harvested in 5 ml of 0.02% (*v/v*) Tween 80 after the co-culture times to be evaluated (two, three or four days). The total cells’ suspension volume was equally distributed into five MM-selection-agar plates supplemented with 200 μg/ml cefotaxime and variable concentration of phleomycin (25, 50 and 100 μg/ml) to inhibit the growth of *A. tumefaciens* and select for the positive *A. sojae* transformants, respectively. The plates were incubated in the dark at 28°C until growth of fungal colonies was observed.

### Extraction of genomic DNA

Genomic DNA (gDNA) from *A. sojae* was extracted as described by Melo et al. [13] with some modifications. A mixture of 100 mg of fungal growth (either mycelia or spores from PDA), one volume of lysis buffer (1 mM Na_2_EDTA, 100 mM NaCl, 1% (*w/v*) SDS, 2% (*v/v*) Triton X-100 and 10 mM Tris, pH 8.0) and one volume of glass beads. (212–300 μm; Sigma, Germany) was vortexed for 5 min for breaking of fungal cells. The released gDNA was then isolated with a phenol-chloroform mixture according to standard procedures [14].

### Gene expression analysis

#### PCR analyses

The T-DNA integration in the fungal genome of randomly selected transformants was assessed by PCR. The specific primers sets BLE-F/BLE-R and EGFP-F/EGFP-R (Table 1) were used to target the *ble* and the *egfp* gene located in the T-DNA region, respectively. Genomic DNA from untransformed *A. sojae* (wild-type; wt) was used as a control.

### Fluorescence microscopy

The expression of EGFP in *A. sojae* transformants was analyzed by fluorescence microscopy. Randomly selected phleomycin-resistant fungal colonies were picked from the selection plates, inoculated on PDA plates supplemented with 100 μg/ml phleomycin, and grown at 28°C for 2–5 days. Fungal growth fractions were immersed in a drop of water on glass slides. The fluorescence signal was visualized using an Axiplan 2 imaging (Zeiss) microscope equipped with a filter set matching the excitation and emission spectra for the EGFP (Ex/Em = 488/509). Images were acquired with the AxioVision (*V. 4.8*) software, setting an exposure time of 900 ms to subtract the background autofluorescent signal of the *A. sojae* wt.

### Western blot

The expression of EGFP in selected transformants was further investigated by Western blot according to standard procedures [14]. The *A. sojae* transformants and wild-type strains were cultured in potato dextrose liquid media for 2–3 days at 28°C on a rotatory shaker at 150 rpm. The mycelia were harvested by filtration, washed with distilled water, dried, and frozen in liquid nitrogen. The frozen mycelia were lyophilized, ground to a fine powder by using a mortar and pestle, and mixed with 2 volumes of distilled water and 3 volumes of Laemmli-sample-loading buffer (100 mM Tris–HCl, pH 6.8, 20% (*v/v*) glycerol, 4% (*w/v*) SDS, 0.2% (*w/v*) bromophenol blue, and 200 mM β-mercaptoethanol). The mixture was boiled at 100°C for 5 min, cooled quickly on ice and then centrifuged at 13,000 × *g* for 5 min. The soluble proteins in the supernatant were separated on a 12% (*w/v*) SDS-PAGE gel and blotted onto a nitrocellulose membrane (Amersham, GE Healthcare) for 2 h at 100 V, by using a wet transblot system (Mini-PROTEAN, Bio-Rad, Germany). Protein transfer was confirmed by staining the membrane with Ponceau S (Roth, Germany). The blots were blocked by incubation for at least 8 h at 4 to 8°C in tris-buffered saline (TBS) containing 5% (*w/v*) nonfat dry milk and 0.1% (*v/v*) Tween 20. Primary and secondary antibodies were diluted with the same buffer. The primary antibody used was rat monoclonal anti-GFP diluted at 1:500 (BioLegend, USA). The blots were incubated with the primary antibody at room temperature for at least 4 h and then washed three times with TBS containing 0.1% (*v/v*) Tween 20. All washing steps were carried out for 10 min. The secondary antibody used was goat anti-rat immunoglobulin G conjugated to horseradish peroxidase (BioLegend, USA) diluted at 1:1,000. The blots were incubated with the secondary antibody at room temperature for at least 2 h, washed three times with TBS containing 0.1% (*v/v*) Tween 20, and then washed once with TBS. The chemiluminescent signal was revealed using ECL substrate and X-ray films for ECL detection (Thermo Scientific, Germany).

### Mitotic stability of transformants

The mitotic stability of the transfected T-DNA was determined in a sample of 20 selected fungal transformants. Fungi were cultured on PDA plates supplemented with 100 μg/ml phleomycin for 4 generations. The stable integration of T-DNA into the genome of the transformants was investigated by PCR analysis, targeting the *ble* and the *egfp* gene with the specific primers sets BLE-F/BLE-R and EGFP-F/EGFP-R, respectively (Table 1).

## Results and discussion

### Phleomycin antibiotic as selectable marker for *A. sojae*

**Figure 2:**
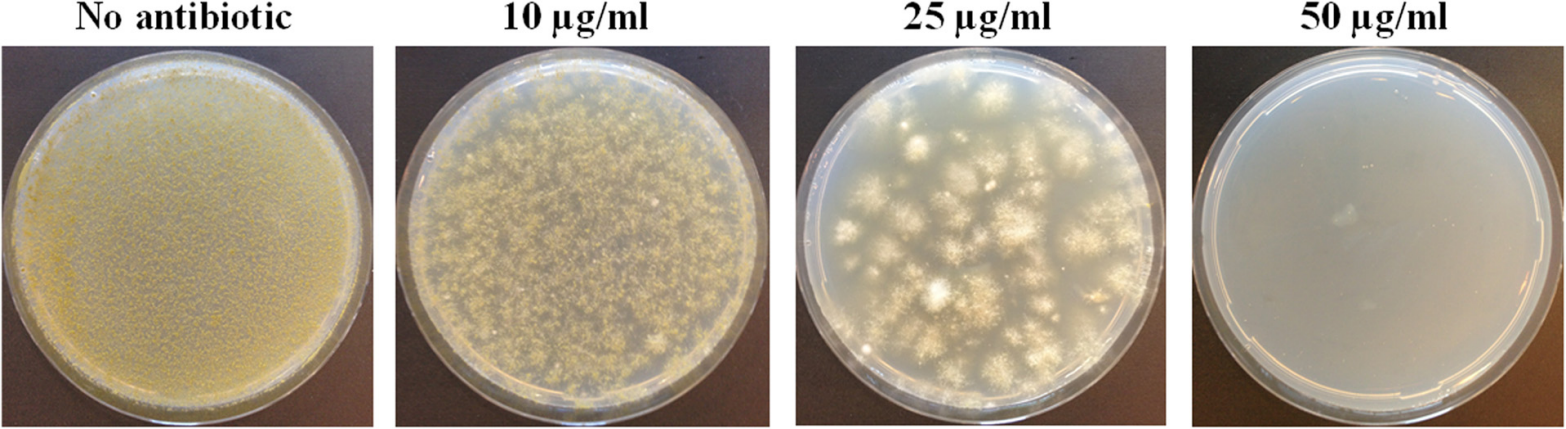
**Phleomycin inhibition.** The *A. sojae* growth on minimal media with various phleomycin concentrations after 7 days incubation. Initial inoculum: 10^5^ spores.

To be able to perform the ATMT procedure with *A. sojae*, the suitability of antibiotics from the bleomycin family was tested as selectable markers for this fungus. Zeocin showed little inhibition for *A. sojae* at concentrations up to 300 μg/ml (data not shown). We thus tested phleomycin, an antibiotic recommended from the manufacturer (InvivoGen, USA) for cells with low sensitivity to zeocin. Our analysis showed that minimal media plates supplemented with 50 μg/ml phleomycin completely inhibited the growth of an *A. sojae*-inoculum of 10^5^ spores for up to 7 days (Figure 2). We thus used this antibiotic concentration for the selection of *A. sojae* transformants in the ATMT.

### Optimization of conditions for ATMT of *A. sojae*

*A. tumefaciens* LBA4404 containing the vector pRM-eGFP was utilized to test the ATMT method. Common ATMT conditions for filamentous fungi [8,12] as mentioned in the methodology were used to verify the viability of the assay. However, in our procedure, the bacterial-fungal co-culture growth was harvested in a 0.02% (*v/v*) Tween 80 solution and the solution equally distributed in several selection plates, contrary to other common ATMT methodologies, where the co-culture growth lying on a membrane is transferred directly onto the selection plates. In preliminary experiments, a concentration of 100 μg/ml of phleomycin was used in the selection plates, aiming to enhance the selectivity of the assay. However, the number of putative fungal transformants was as low as 10 colonies per 10^5^ conidia after three days. Consequently, the concentration of phleomycin in the selection plates was reduced to half, and the number of fungal colonies increased five to eight fold. This result reflected a possible limiting condition where a high concentration of antibiotic affects the survival rate of fresh transformants. On the other side, further reductions in phleomycin concentration led to fungal over-growth, which made the subsequent isolation of single transformants difficult; therefore, selection plates with 50 μg/ml phleomycin final concentration were used for following experiments.

Different co-cultivation times of two, three and four days were assessed in the transformation procedure. An increase of putative fungal transformants was observed on the selection plates with increasing co-cultivation time, ranging from 40 to 100 transformants per 10^5^ conidia from two to four days, respectively. However, co-cultivation times of three days led to the best ratio of positive to false positive transformants. The higher frequency of negative transformants during the four-day co-cultivation was likely due to higher background growth on the selection plates resulting from longer co-cultivation times without antibiotic pressure. We thus used three days of co-cultivation time before selecting the transformants on 50 μg/ml phleomycin in the selection media that yielded approximately 80 putative fungal transformants per 10^5^ conidia.

### Gene expression analysis

#### PCR analysis

**Figure 3:**
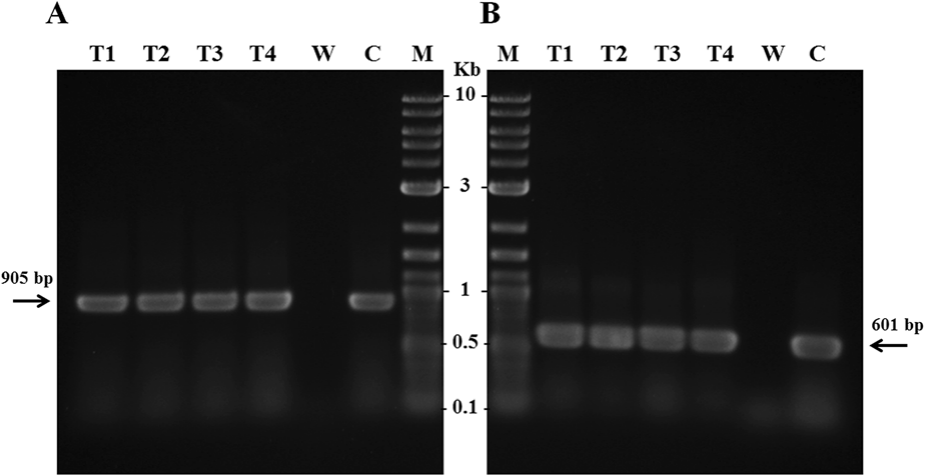
**PCR analysis of** ***A. sojae*** **transformants.** Amplified PCR products from genomic DNA of selected *A. sojae* transformants (T1-T4), confirming the presence of the *ble* gene **(panel A)** and the *egfp* gene **(panel B)**; *A. sojae* wild-type sample used as negative control (W); pRM-eGFP vector used as positive control (C); molecular size marker (M).

Putative fungal transformants were examined by PCR analysis to confirm successful chromosomal integration of the T-DNA. The genomic DNA from selected *A. sojae* transformants and the wild-type as negative control were used to target *ble* and *egfp* genes with the specific primers sets BLE-F/BLE-R and EGFP-F/EGFP-R, respectively (Table 1). PCR products of expected sizes of 905 bp for the *ble* gene and 601 bp for the *egfp* gene were obtained from the transformants but not from the wild-type DNA (Figure 3). Subsequent sequencing analysis of the PCR products confirmed the presence of both genes in the *A. sojae* transformants.

### Fluorescence microscopy

**Figure 4:**
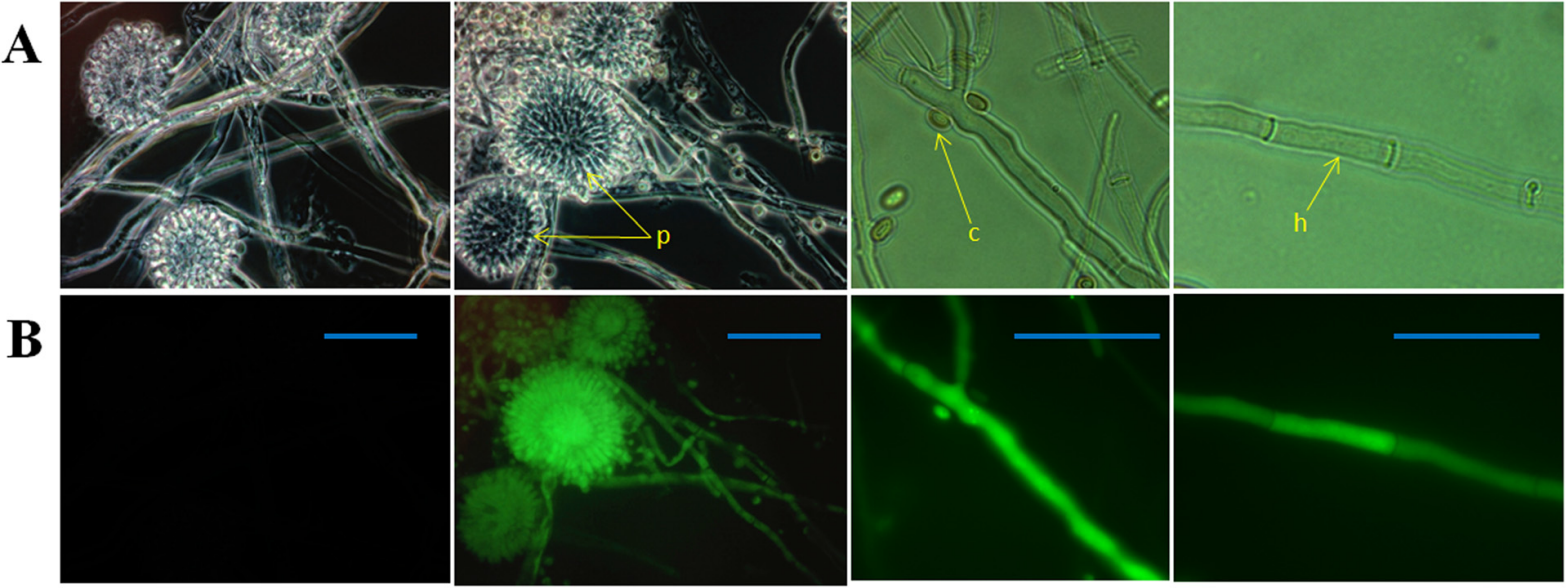
**Fluorescence microscopy analysis of** ***A. sojae*** **wild-type and selected transformants with EGFP.** Highlighted with arrows are hyphae (h), conidiophores (p) and conidia (c) of fungal transformants showing fluorescent signal. **A** and **B** are bright field and fluorescence images, respectively. Scale bar = 50 μm.

Fluorescence microscopy studies were carried out to verify the expression of the EGFP reporter gene in selected *A. sojae* transformants. The fungal transformants showed regular fluorescent signal in structures like hyphae and conidiophores but in isolated conidia the signal was scarce (Figure 4). Moreover, the intensity of the fluorescent emission appeared to be higher in growing hyphal tips and conidiophores. The wild-type strain showed some autofluorescent background signal, which was considerably less compared to the transformants. Therefore, an exposure time of 900 ms was used in the microscopy analysis, where no fluorescent signal was detected in the wild-type but in the transformants (Figure 4). These results indicate that the transformants were able to express the EGFP gene, and validate the PCR results, where integration of the EGFP gene was demonstrated. This analysis facilitated the selection of positive transformants vs. wild-type, as there was no noticeable morphological difference between the colonies of these fungi on the agar plates.

### Western blot analysis

**Figure 5:**
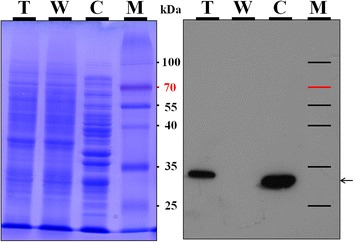
**Western blot.** Intracellular protein profiles (left panel) and Western Blot analysis (right panel) of *A. sojae* transformant (T), wild-type (W), and control GFP-overexpressing *E. coli* (C) strains. Molecular size marker (M).

Western blot analysis was performed to confirm the presence of EGFP in *A. sojae* transformants. Intracellular fungal extracts were electrophoresed under denaturing conditions (12% (*w/v*) SDS-PAGE) (Figure 5). Prior antibody hybridization, Ponceau S staining was performed to verify the transfer of intracellular proteins onto the nitrocellulose membrane. After Western blotting with anti-GFP (rat) primary and anti-rat IgG-horseradish peroxidase conjugate secondary antibodies, chemiluminescent signals in form of unique bands were detected in the fungal transformant and the positive control (Figure 5). These bands were shown at the proximate 26.9 kDa molecular mass expected for the EGFP. No visible band was detected in the wild-type sample. These results indicate that EGFP protein had been produced in the *A. sojae* transformants intracellularly and corroborate the fluorescence microscopic analysis, where EGFP expression was detected. Furthermore, the suitability of PgpdA for heterologous gene expression in *A. sojae* was demonstrated.

### Mitotic stability of transformants

The mitotic stability of the T-DNA in *A. sojae* transformants was examined by subculturing them under phleomycin pressure aiming to increase the stability of the recombinant DNA. Previous experiments with no antibiotic in the media showed little T-DNA retention in the fungal transformants. Therefore, a relatively high concentration of phleomycin was used in the media to favor the survival of the most stable transformants. Twenty *A. sojae* transformants were subcultured for four generations on PDA plates supplemented with 100 μg/ml phleomycin. After four generations all the transformants were screened by PCR for the presence of the *ble* and *egfp* genes. Both genes were PCR-amplified in 8 of the 20 transformants, corresponding to a 40% mitotic stability of genomic T-DNA integration. Unique amplification of either the *ble* or the *egfp* gene was not observed in any of the samples, thus discarding any partial integration of the T-DNA cassette. The negative transformants were further examined with the fluorescence microscope to check for EGFP expression. As expected, none of these fungal samples showed a fluorescent signal. These results indicate that the T-DNA was aborted in these fungi, and suggest that a resistance mechanism different from the *ble* gene played a role in the tolerance of these fungi to phleomycin.

It is known that transformation efficiencies in filamentous fungi are generally low [15]. In fungi like *A. oryzae* and *A. nidulants,* high abortive rates of transformed DNA were reported. For example, an abortive rate of the *A. nidulants argB* gene in *A. oryzae* was evidenced in approximately 90% of the initial transformants [16,17]. Filamentous fungi with multinucleate conidia have been considered a probable cause for recalcitrant transformation and thus conidia with one nucleus are commonly considered to be useful in mutagenesis approaches [18]. To test for multiple nuclei we used DAPI-staining on spores of the *A. sojae* ATCC 20235 used in this study and found that its conidia contain multiple nuclei ranging in numbers from 1 to 6 (data not shown). Possibly, the abortive transformations we observed in ATCC 20235, is caused by insufficient T-DNA integration in all nuclei; for instance a single T-DNA locus could lead to mitotically unstable transformants. However, the exact mechanism resulting in abortive events in this fungus is not well-known. More studies about the reproductive cycle of this fungus are needed to explain this phenomenon. Nevertheless, the ATMT method described in this study demonstrated to be useful to produce *A. sojae* transformants, and our analysis showed that these transformants were able to successfully express the heterologous EGFP protein using the constitutive promoter PgpdA.

## Conclusions

We achieved successful transformation of *A. sojae* through the established ATMT protocol using *ble* as a selective marker. The fungal transformants were remarkably less sensitive to phleomycin in comparison with the wild-type and showed expression of the reporter gene EGFP, indicating successful chromosomal integration of the T-DNA. This transformation system should facilitate further protein expression studies in this fungus and may be applicable to other phleomycin-sensitive filamentous fungi.

## Abbreviations

ATMT: *Agrobacterium tumefaciens*-mediated transformation
EGFP: Enhanced green fluorescent protein
gDNA: Genomic DNA
LB: Left border of T-DNA (transfer DNA)
PCR: Polymerase chain reaction
RB: Right border of T-DNA (transfer DNA)
